# Ménière’s disease and vestibular migraine: a narrative review of pathogenetic insights, diagnostic evolution, and clinical management advances

**DOI:** 10.3389/fneur.2025.1653509

**Published:** 2025-10-14

**Authors:** Hongwei Sun, Gang Zhang, Yingxin Zhang, Tong Li, Xiuzhen Du, Zhensheng Fang

**Affiliations:** ^1^Shandong First Medical University and Shandong Academy of Medical Sciences, Jinan, Shandong, China; ^2^Department of Otolaryngology, The Second Affiliated Hospital of Shandong First Medical University, Taian, Shandong, China; ^3^Department of Gynaecology, The Second Affiliated Hospital of Shandong First Medical University, Taian, Shandong, China

**Keywords:** Ménière’s disease, vestibular migraine, endolymphatic hydrops, biomarkers, genetic factors

## Abstract

Ménière’s disease (MD) and vestibular migraine (VM) are two common vestibular disorders with significant clinical overlap in their symptomatic presentations, including vertigo, hearing loss, tinnitus, and aural fullness. Although distinct diagnostic criteria exist for each, this symptomatic similarity often makes early-stage differentiation challenging. While recent studies have found potential biomarkers for MD and VM, their diagnostic utility remains limited by small sample sizes and lack of standardized validation protocols. This necessitates continued reliance on a synthesis of established guidelines (e.g., from the Bárány Society), detailed analysis of symptom temporal profiles, and ancillary examinations. This review presents a comparative analysis of the pathogenesis, clinical characteristics, and diagnostic criteria of MD and VM, summarizes recent research advances, and proposes key directions for future investigation. Major priorities include: (1) applying single-cell transcriptomics and genetically engineered animal models to further elucidate disease mechanisms underlying MD and VM; (2) establishing imaging-based specific biomarkers through high-resolution inner ear MRI; (3) validating candidate serum biomarkers using standardized proteomic platforms; and (4) integrating clinical features, imaging findings, and molecular biomarkers via machine learning approaches to improve diagnostic accuracy and enable personalized treatment strategies.

## 1 Introduction

Ménière’s disease (MD) and Vestibular Migraine (VM) are two clinically prevalent vertigo-associated disorders (1). They are characterized by overlapping symptoms such as episodic vertigo, tinnitus, and aural fullness, which create substantial diagnostic dilemmas in clinical practice. Endolymphatic hydrops (EH) is the defining pathological hallmark of MD, whereas its role in VM remains controversial and is considered nonspecific when present (1–3). However, current research predominantly focuses on single-disease mechanisms or isolated clinical feature analyses, lacking integration of studies on their molecular and cellular intersections and genetic heterogeneity, leading to diagnostic criteria reliant on subjective symptom clusters. Furthermore, as a nonspecific pathological manifestation, the dynamic evolution of EH in both disorders remains poorly characterized. This review conducts a multidimensional comparative analysis of pathological mechanisms, genetic profiles, clinical manifestations, and diagnostic criteria to systematically delineate the core disparities between MD and VM. Concurrently, by integrating the application potential of cutting-edge technologies such as whole-genome sequencing and intratympanic gadolinium-enhanced MRI, proposing that future research should employ multi-omics approaches and precision medicine strategies to elucidate the distinct pathogenic mechanisms underlying each disorder and to discover objective biomarkers. This will refine diagnostic differentiation models, thereby offering novel perspectives to overcome current clinical diagnostic bottlenecks.

## 2 Differences at cellular and molecular levels in pathogenesis

### 2.1 Inner ear cellular level

Ménière’s disease (MD) is a common otogenic vertigo disorder characterized by endolymphatic hydrops (EH) in the inner ear. The exact pathogenesis of MD remains unclear. Hallpike and Cairns were the first to identify EH as the characteristic pathological change in MD through post-mortem examination (4). However, subsequent research by Chen Zi et al. (5) found that EH is not the direct cause of MD. Potential causes leading to EH may include autoimmune diseases, genetic factors, inner ear circulatory ischemia, dysregulation of salt-regulatory hormones, viral sequelae, and inflammatory reactions. The pathophysiological mechanisms of vestibular migraine (VM) are also not fully understood and are widely thought to be associated with ion channel defects, cortical spreading depression, inflammation, and genetic predisposition (6).

In VM patients, channels controlling ion flow in the brain (such as potassium channels) exhibit intermittent functional defects. When stimulated by triggers like stress or hormonal fluctuations, these channels open abnormally, causing large amounts of intracellular potassium ions to leak into the extracellular fluid, significantly increasing local potassium ion concentration. The elevated potassium ions stimulate trigeminal nerve endings surrounding the pial arteries, leading to an imbalance in nerve cell membrane potential and triggering abnormal electrical signals. The activated nerve endings release neuropeptides such as Substance P and Calcitonin Gene-Related Peptide (CGRP), causing abnormal cerebral vascular dilation, increased vascular wall permeability, and neurogenic inflammation. These responses stimulate trigeminal nociceptors, resulting in headache. Simultaneously, released inflammatory mediators like CGRP act on the inner ear via the bloodstream, causing changes in inner ear vascular permeability and microcirculatory disturbances, interfering with hair cell signal transduction, and ultimately leading to rotational vertigo (7).

For patients diagnosed with MD, studies have confirmed that vestibular endolymphatic hydrops is a prerequisite for MD vertigo attacks (8). Okuno et al. (9) conducted a histopathological study on the localization, frequency, and severity of EH in 22 temporal bone specimens from 16 MD patients. They found that in 17 specimens, the saccular membrane bulged laterally into the vestibular scala, while in 2 specimens, Reissner’s membrane at the helicotrema of the cochlear basal turn bulged upward into the vestibular scala, occupying most of the vestibular perilymphatic space. Their results indicated that EH is mostly located in the inferior vestibule, with the highest severity occurring in the saccule. Furthermore, the degree of hydrops in the cochlear apical turn was significantly higher than in other regions. Li et al. (10) performed bilateral intratympanic gadolinium contrast injection on 178 patients diagnosed with unilateral MD. They detected symptomatic EH in all affected ears, but the incidence and degree of EH in the cochlea of the affected ear were significantly higher than in the vestibule. The direction and severity of EH decreased progressively from the cochlea toward the vestibule, similarly demonstrating Okuno et al.’s conclusions.

Regarding the specific cellular mechanisms of the imbalance between endolymph production and absorption, studies have indicated that dysfunction of the stria vascularis (SV) may disrupt ionic homeostasis, further contributing to EH in the progression of MD (11). Various changes in inner ear physiology, such as pressure fluctuations, ionic imbalance, and alterations in cochlear potentials, are potential mechanisms of MD (12–14). It has been reported that because hair cells are metabolically active and highly dependent on blood supply, hypoxia resulting from insufficient inner ear blood supply reduces their metabolic efficiency. This leads to sodium ion retention in the endolymph, increasing endolymph osmotic pressure, which causes water to seep from the perilymphatic space into the endolymph, resulting in EH. Other research suggests that degeneration or dysplasia of the endolymphatic sac leads to abnormal dilation of the endolymphatic space, impairing absorption function and ultimately causing EH (15).

EH is recognized as the pathognomonic pathological hallmark of MD. However, its detection is not clinically pathognomonic, as EH may also present as an incidental or secondary finding in other inner ear disorders, such as sudden sensorineural hearing loss, otosclerosis, and vestibular migraine. Therefore, the clinical interpretation of EH must be integrated with the full clinical picture to avoid misdiagnosis.

### 2.2 Imaging findings and serum biomarkers

To clarify the correlation between MD, VM, and EH, researchers performed bilateral intratympanic gadolinium injection in patients with definite unilateral MD, followed by MRI 24 h later to evaluate the presence and grading of EH. The results showed that all patients exhibited varying degrees of EH in the vestibular and/or cochlear regions. Among patients diagnosed with or suspected of having VM, some also showed EH on MRI (2). To further distinguish between MD and VM using imaging, another study compared VM and MD patient groups by performing MRI scans 4 h after intravenous injection of a single dose of gadobutrol (1.0 mmol/mL), assessing cochlear endolymphatic hydrops (CEH), vestibular endolymphatic hydrops (VEH), and asymmetric perilymphatic enhancement (PLE). The results demonstrated that none of the VM patients exhibited enhancement in CEH, VEH, or PLE, while the MD group showed significant enhancement. This allows for differential diagnosis between MD and VM in appropriate clinical settings (3). In conclusion, the occurrence of EH in VM patients is considered coincidental, and inner ear gadolinium-enhanced MRI can reveal the presence and severity of EH, thereby providing important objective imaging evidence for distinguishing MD from VM.

Beyond radiological differences, distinct molecular biomarkers further support the pathological divergence between MD and VM. In recent studies, Wu et al. analyzed MD-related data from the GEO database using bioinformatics and machine learning. The results indicated that CD5 and AJUBA are potential biomarkers for MD, while resting T cells, memory T cells, activated T cells, and dendritic cells are core immune cells involved in MD (16). Lin et al. compared samples from 15 patients and healthy controls, revealing significant differences in the protein expression profiles of MD patients. They further confirmed the expression of CHMP1A and MMP9 in the endolymphatic sac of patients and the inner ear of mice, suggesting that CHMP1A, VPS4A, FCN3, and MMP9 could serve as potential biomarkers for MD (17). Demartini et al. analyzed the expression characteristics of biomolecules such as CGRP, inflammatory factors, and endocannabinoids using clinical and preclinical research from the PubMed/MEDLINE database, investigating biomarkers in the body fluids of VM patients. The results showed that CGRP levels fluctuate and remain elevated during both VM attacks and interictal periods, suggesting its potential as a diagnostic marker (18).

Moreover, recent single-cell transcriptomic studies have elucidated the distinction between MD and VM from the perspective of immune mechanisms. VM patients exhibit a high degree of overlap with migraine (MI) patients in the transcriptional profiles of innate immune cells such as natural killer (NK) cells, leading to their classification within a shared immune cluster (MI + VM cluster). This cluster demonstrates a Type 1 innate immune cell-polarized response, characterized by the release of cytokines including interleukin (IL)-12, IL-15, and IL-18, driving a dominant Type 1 lymphoid immune response pattern. In contrast, MD presents a markedly different immunopathological profile. Single-cell RNA sequencing (scRNA-seq) data of monocytes from MD patients revealed two distinct clusters: one “inactive cluster” and another “monocyte-driven cluster.” The unique pathways activated in the latter cluster involve responses to biotic stimuli, which stands in sharp contrast to the metal ion response pathways observed in the VM/MI cluster. These immunological distinctions provide compelling evidence that MD and VM/MI are independent disease entities with fundamentally distinct pathogenic mechanisms (19).

### 2.3 Genetic and molecular-level mechanisms

Current research on MD at the genetic and molecular level falls into two categories: first-generation sequencing and next-generation sequencing (NGS). Studies primarily focus on candidate genes validated in familial Meniere’s disease (FMD) pedigrees and reported candidate genes in sporadic Meniere’s disease (SMD) (20). With the evolution of gene sequencing technology, first-generation sequencing for FMD research has progressed to NGS. Its rapid development enables whole-exome sequencing (WES) in MD patients, allowing comparison and filtering of genetic variants against healthy individuals with similar genetic backgrounds, thereby enhancing the reliability of identifying MD-associated pathogenic genes.

Using NGS and advanced bioinformatics tools, international researchers have identified novel variant genes such as OTOG, LSAMP, PRKCB, DTNA, DPT, SEMA3D, and FAM136A (21). Domestic researchers further proposed EGFLAM and ITGAS as additional candidate pathogenic genes (22). However, validation in large-scale cohorts and other pedigrees remains limited, and direct evidence from cellular or animal models is still lacking. Previous SMD genetic studies primarily centered on genes related to water channels (e.g., KCNE, AQP) and immune responses (e.g., HLA).

Although extensive genetic research has not yet established a direct link between MD and single or few genes, advancements in whole-genome sequencing (WGS), combined with rigorous screening of large clinical cohorts and optimized multidimensional data analysis, hold promise for identifying MD susceptibility loci. This could provide genetic insights for clarifying pathogenic mechanisms.

VM also exhibits genetic predisposition. Studies indicate associations between VM and genes such as SCN1A, SLC1A3, PBMC gene expression profiles, Egr1 (early growth response gene 1), CAV1, KCNA1, TRPM7, CACNA1A, and ATPlA2 (23). These genes differ significantly from those linked to MD. Understanding these genetic correlations could improve VM diagnosis accuracy, reduce misdiagnosis, and guide the development of targeted VM therapies.

**Figure 1:**
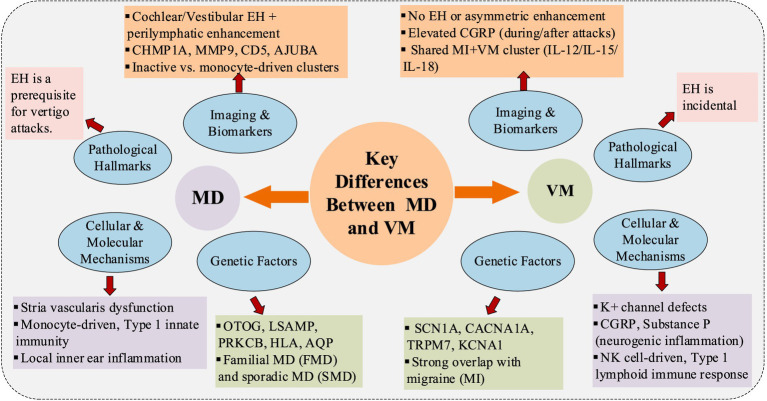
Comparison of the pathogenesis between MD and VM.

In summary, this section elucidates the differences between MD and VM at cellular, molecular, and genetic levels. As illustrated in Figure 1, the key distinctions can be summarized as follows: EH serves as the characteristic pathological hallmark of MD but lacks specificity in VM, where ion channel dysfunction and neurogenic inflammation predominate. Immune profiling via single-cell RNA sequencing reveals divergent pathways - MD exhibits monocyte-driven responses while VM demonstrates type 1 innate immune responses. At the genetic level, MD-associated genes differ significantly from VM-linked loci, supporting their distinct etiopathogenic mechanisms.

## 3 Clinical manifestations

Although MD and VM share many similar clinical symptoms, there are currently no highly specific indicators to distinguish between them. Nevertheless, relative distinguishing features can assist clinicians in making initial diagnoses.

Regarding headache and migraine-related symptoms, most VM patients experience unilateral pulsating headaches, but approximately 30% of cases exhibit isolated vertigo without headache (23–25), with at least one migraine-related feature such as photophobia, phonophobia, or visual aura. In contrast, the prevalence of migraine among MD patients varies widely, ranging from 2 to 80% (26–28). Some studies suggest its incidence is significantly higher than in healthy populations, while others indicate no significant difference between these groups (29, 30). In summary, the most sensitive and unique feature of VM is recurrent moderate-to-severe migraine (31, 32). This indicates that migraine is a core symptom of VM, easily distinguishable from MD.

Although the presence of migraine serves as a key feature for distinguishing between VM and MD, clinical differentiation remains challenging primarily because MD attacks themselves may trigger migraine-like symptoms, thereby confounding the clinical presentation of VM. Louisa Murdin et al. selected 123 patients with a history of migraine. By inducing vertigo through rotational/caloric vestibular testing in 79 patients and comparing them with 44 control subjects, the incidence of migraine attacks within 24 h was assessed. The results showed that 49% of the experimental group experienced a migraine within 24 h, compared to only 5% in the control group. This suggests that provoked vertigo can act as a trigger for migraine, although benign paroxysmal positional vertigo (BPPV), MD, and other conditions may also potentially trigger migraines (33). Existing studies have indicated that during attacks, MD patients exhibit sensitivity to at least two external stimuli (such as photophobia, phonophobia, or visual aura), and some MD patients without a history of migraine also experience migraines during their attacks (26).

Current research widely recognizes that the duration, frequency, and characteristics of vertigo episodes are the primary factors for differential diagnosis between MD and VM. A comparison of these factors is presented in Table 1 (26, 31–36).

**Table 1:** Comparison of triggering factors for vertigo attacks.

Features	VM	MD
Duration	Seconds to days (primarily minutes to hours)	Typically lasts 20 min to 12 h (median duration: 2–4 h)
Attack frequency	≥1/month (Higher frequency in MD with VM comorbidity, *p* < 0.01)	Fluctuating (May increase to weekly/monthly frequency during progression)
Triggering factors	Head position change, visual motion 20–85% with motion intolerance	Mostly spontaneous, occasionally triggered by stress/dietary factors
Subjective description	Spinning, floating, imbalance sensation	Intense spinning sensation with nausea/vomiting

As shown in Table 1, vertigo in MD and VM exhibits significant differences in duration and frequency. Typical vertigo episodes in MD patients usually last several hours with relatively fixed duration. Neff et al. (32) confirmed a significant statistical association between vertigo lasting hours and MD (*p* < 0.05). In contrast, vertigo in VM patients varies widely in duration, ranging from seconds to days (35). Wang et al. (34) noted that monthly episode frequency also differs significantly between the two diseases and serves as an important indicator for distinguishing MD from VM. The vertigo episode frequency is relatively low in pure MD patients, while MD patients with comorbid VM have significantly higher monthly vertigo frequency (*p* < 0.01). Subjective descriptions also differ markedly: VM patients typically report that changes in position, head movements, or visual motion trigger or worsen vertigo, manifesting as sensations of spinning, floating, swaying, or imbalance (35). Research (36) found that a large proportion of VM patients exhibit head-motion intolerance, positional vertigo, or spontaneous vertigo during episodes. Unlike VM, MD patients most commonly present with spontaneous vertigo (37) and fluctuating hearing loss during attacks. Study (23) indicates that progressive hearing decline is the most diagnostically sensitive and clinically recognizable manifestation of MD. Lopez-Escamez et al. suggested that MD patients can determine the presence of hearing loss through subjective symptoms and hearing tests, whereas VM shows the opposite pattern (38).

In summary, the hearing loss in VM is typically mild, readily reversible, and low-frequency, often accompanied by tinnitus and aural fullness (39). This contrasts sharply with the distinct, progressive, and often disabling hearing loss that is characteristic of MD (40, 41). Therefore, the nature and presence of hearing loss serve as a crucial diagnostic feature for distinguishing between these two disorders.

## 4 Diagnostic criteria

The pathogenesis of MD and VM exhibits significant differences at the cellular and molecular genetic levels of the inner ear, with distinct clinical manifestations. To differentiate between MD and VM, scholars and organizations have developed specific diagnostic criteria based on their clinical presentations. These criteria continue to evolve, with MD standards varying across countries yet sharing similarities alongside notable differences (42–44). Diagnostic criteria for MD from different countries are summarized in Table 2.

**Table 2:** Diagnostic criteria for Meniere’s disease from different countries.

Criteria	2015 Bárány society	2017 Chinese guidelines	2020 US guidelines	2017 Japanese criteria
Vertigo episodes	≥2 episodes (spontaneous)	≥2 episodes (spontaneous)	≥2 episodes (spontaneous)	≥2 episodes (spontaneous)
Duration per episode	20 min– 12 h	20 min – 12 h (Definite MD) 20 min – 24 h (Probable MD)	20 min – 12 h (Definite MD) 20 min – 24 h (Probable MD)	10 min – Several hours
Hearing loss requirement	Low/mid-frequency SNHL	Low/mid-frequency SNHL (mandatory)	Low/mid-frequency SNHL (mandatory)	Low/full-frequency SNHL (mandatory)
Fluctuating cochlear symptoms	At least one of three:(tinnitus/ear fullness/fluctuating HL)	All three mandatory (Definite MD)	All three mandatory (Definite MD)	All three mandatory (Typical MD) Cochlear/vestibular types (Atypical MD)
Imaging requirement	Not mandatory	Not mandatory	Not mandatory	MRI-confirmed endolymphatic hydrops (mandatory)
Exclusions	Other vestibular disorders	12 diseases (e.g., sudden deafness)	Other causes via clinical trial	Diseases beyond CN VIII disorders
Secondary endolymphatic hydrops	Must exclude	Must exclude	Must exclude	Must exclude
Atypical MD subtypes	Not defined	Not defined	Not defined	Cochlear/vestibular types
Vestibular dysfunction evidence	Not required	Not required	Not required	Peripheral signs required (e.g., nystagmus)
Symptom-test temporal link	Vertigo-HL association required	Vertigo-HL association required	Vertigo-HL association required	Vertigo period must show HL fluctuation

In 1972, the American Academy of Otolaryngology-Head and Neck Surgery (AAO-HNS) issued guidelines classifying MD, defining core symptoms as recurrent vertigo (≥2 episodes lasting 20 min to 24 h), fluctuating hearing loss (predominantly low-frequency), tinnitus, and/or aural fullness. The classification included “definite” and “possible” diagnoses but relied solely on clinical history without objective testing, posing limitations. The 2015 AAO-HNS revision eliminated the “definite” category, retaining only “confirmed” and “probable” diagnoses. It shortened vertigo duration per episode, adjusted hearing criteria, and added exclusion requirements (e.g., vestibular migraine and central vertigo) (45).

In 2015, the Barány Society updated its criteria (based on AAO-HNS 1995), emphasizing the association between episodic vestibular symptoms and hearing loss: specifically, requiring two typical vertigo episodes + hearing loss (at least one low-frequency deficit) + tinnitus/aural fullness (37). It also recommended MRI to exclude structural abnormalities like acoustic neuromas.

Guideline for the Diagnosis and Treatment of Meniere’s Disease (46), referencing international Barány consensus while incorporating local data, adjusted diagnostic criteria, auxiliary tests, and exclusions. It mandated tinnitus/aural fullness for diagnosis and emphasized low-frequency sensorineural hearing loss via pure-tone audiometry. Gadolinium-enhanced MRI for endolymphatic hydrops was recommended only for complex cases. Exclusion diagnostics required temporal bone CT or surgical exploration to rule out perilymphatic fistula.

Japan’s 2017 revised MD criteria (47) listed inner ear MRI as optional support (non-mandatory), mandated concurrent tinnitus, aural fullness, and fluctuating hearing loss, and refined classification into “typical/atypical MD” and “bilateral MD”—the latter requiring exclusion of systemic diseases.

The AAO-HNS 2020 Clinical Practice Guideline for MD (48) refined criteria by specifying vertigo as spontaneous rotational vertigo. It introduced “confirmed” and “suspected” diagnostic pathways, mandating MRI to exclude other pathologies while suggesting selective use for central lesion exclusion.

In contrast, VM diagnostic criteria achieved global consensus among researchers. However, VM’s journey from proposal to inclusion in ICHD-3 spanned multiple stages. The 2012 joint Barány Society and International Headache Society (IHS) criteria first defined VM as a distinct entity, requiring vestibular symptoms and migraine association (“definite VM”) or partial links (“probable VM”). ICHD-3 beta (2013) adopted Barány’s framework but added ≥5 episodes and exclusion of other vestibular disorders. Barány’s 2018 update expanded symptoms to include motion sensitivity and visually induced dizziness, allowing non-simultaneous vertigo and headache. Current international standards (ICHD-3, 2018; revised 2019) are detailed in Table 3 (42–44).

**Table 3:** Diagnostic criteria for vestibular migraine (VM).

Classification	Item	Specific criteria	Notes/Supplement
Definite VM	A	≥5 episodes meeting criteria C and D	Requires frequency threshold
	B	History of migraine or current episodes fulfilling ICHD-3 criteria for migraine without aura or migraine with aura	Requires historical or current migraine evidence
	C	Moderate-to-severe vestibular symptoms lasting 5 min to 72 h	Moderate: Affects daily activities but manageable Severe: Incapacitating
	D	≥50% of episodes associated with ≥1 migraine feature: 1. Headache features (≥2 of): (1) Unilateral; (2) Pulsating; (3) Moderate–severe; (4) Aggravated by activity 2. Photophobia or phonophobia 3. Visual aura	Requires clear symptom linkage
	E	Not better accounted for by other ICHD-3 diagnoses or vestibular disorders (e.g., MD, BPPV)	Exclusion via differential diagnosis
Probable VM	A	≥5 episodes of moderate-to-severe vestibular symptoms (5 min–72 h)	Same as Definite VM Criterion C
	B	History of migraine or migraine features during attacks	Definite VM requires B + D; Probable VM requires only one of these
	C	Not better accounted for by other ICHD-3 diagnoses or vestibular disorders	Same as Definite VM Criterion E
Vestibular symptom classification	A	Spontaneous vertigo: Internal vertigo (self-motion illusion/impairment) or external vertigo (environment-spinning/falling)	Non-triggered episodes
	B	Positional vertigo: Induced by head-position changes	Differentiate from BPPV
	C	Visually induced vertigo: Triggered by moving visual stimuli (e.g., traffic, crowds)	Static patterns (e.g., fixed images) excluded
	D	Head-motion-induced vertigo: Provoked by active head movement	Exclude cervicogenic vertigo
	E	Head-motion-induced dizziness with nausea: Spatial disorientation-type dizziness only	Other dizziness types (e.g., lightheadedness) excluded

## 5 Treatment strategies

### 5.1 Treatment strategies for MD

MD currently cannot be clinically cured. Existing therapeutic strategies primarily focus on symptom control, with the optimal treatment goals being to terminate vertigo attacks, eliminate tinnitus, and reverse hearing loss. The treatment priorities include alleviating acute-phase vertigo symptoms, preventing recurrence, and achieving long-term control of progressive inner ear dysfunction. Although certain therapeutic effects have been achieved in the first two priorities, significant challenges remain in controlling the progressive dysfunction of the inner ear in the long term. Current treatment approaches primarily involve pharmacological and surgical interventions, accompanied by certain lifestyle modifications.

#### 5.1.1 Pharmacotherapy for MD

The core objectives in the acute phase of MD are rapid termination of vertigo and alleviation of aural fullness, commonly achieved through pharmacological interventions such as vestibular suppressants and corticosteroids.

Centrally acting antihistamines such as dimenhydrinate, meclizine, and prochlorimethazine inhibit vestibular system activity via anticholinergic mechanisms while providing antiemetic effects (49, 50). Benzodiazepines such as diazepam and lorazepam suppress vestibular nuclear neuronal discharge by activating GABA receptors (51).

Diuretics including hydrochlorothiazide, acetazolamide, and chlorthalidone serve as first-line treatments for MD, reducing vertigo attack frequency by regulating endolymphatic fluid balance (52, 53).

Betahistine is used in multiple European and Asian countries to improve inner ear microcirculation, with a standard dosage of 48–96 mg/d (54). High-dose short-term administration (288–480 mg/d) may be employed for refractory cases, though it is excluded from routine treatment in the United States due to lack of FDA approval (55).

Intratympanic steroid injections (ITS), as a second-line therapy, significantly improve vertigo frequency and severity. Common protocols include daily dexamethasone (4 mg/mL) for 5 consecutive days or weekly injections for 1–4 weeks (56, 57). Alternatively, two methylprednisolone (62.5 mg/mL) injections administered biweekly also demonstrate safety and efficacy comparable to gentamicin (58, 59).

Intratympanic gentamicin injection (ITG), a fourth-line intervention, controls vertigo by chemically ablating vestibular function, achieving an 87–90% reduction in attacks with a single administration (53, 60). However, its ototoxicity may cause hearing loss, particularly irreversible deafness in individuals with unscreened MTRNR1 gene mutations, necessitating comprehensive informed consent prior to treatment (59).

All clinical pharmacotherapeutic regimens exhibit limitations, requiring individualized selection based on contraindications and side effects.

#### 5.1.2 Surgical intervention for MD

In terms of surgical approaches, although procedures such as endolymphatic sac decompression (ESD) and vestibular neurectomy (VN) can provide high vertigo control rates in some refractory cases (61, 62), factors including surgical invasiveness, complication risks (e.g., hearing damage, cerebrospinal fluid leakage), and insufficient long-term data for certain techniques limit their widespread adoption.

ESD remains the preferred surgical option for early-stage refractory MD due to its straightforward technique and low complication rate, effectively preserving hearing and vestibular function. Literature (60) indicates vertigo control rates of 64.5–90% with potential to delay hearing loss progression. However, the long-term efficacy of ESD remains controversial, with some scholars suggesting its effects may relate to placebo response, and it lacks support from double-blind trials.

Recently, endolymphatic duct blockage (EDB) has emerged as a novel surgical technique demonstrating advantages in vertigo control. Saliba et al.’s comparative study (62) showed a 96.5% complete vertigo control rate at 24 months post-EDB, significantly higher than the 37.5% in the ESD group, with stable hearing levels and improved quality of life in both cohorts.

As a destructive procedure, VN achieves significant vertigo control (approaching 100% in indicated patients (55)), suitable for intractable MD patients with poor residual hearing. Studies by Yu et al. (55) found comparable outcomes in vertigo control and quality of life improvement between VN and cerebellectomy groups. However, potential complications including meningitis, cerebrospinal fluid leakage, or epidural hematoma have led to its declining clinical use.

Overall, current international guidelines prioritize pharmacological and intratympanic interventions as first-line therapies, reserving surgery as a last resort after failed conservative treatment, mandating rigorous assessment of clinical indications and risk–benefit ratio (55–62).

#### 5.1.3 Lifestyle modifications

Long-term management of MD can be directed toward multidimensional approaches, employing stepped-care therapy regimens such as salt-restricted diets, intratympanic injections, and vestibular rehabilitation to reduce the frequency of disease attacks, thereby slowing the progression of hearing decline. Specifically: improve sleep quality (63), screen and intervene for obstructive sleep apnea-hypopnea syndrome (64), and establish regular routines to reduce vestibular load; strictly limit sodium intake (low-salt diet), and avoid inner ear irritants such as alcohol, caffeine, and tobacco (65); reduce psychological stress through environmental adjustments to disrupt the “stress-vertigo” vicious cycle. On this basis, vestibular rehabilitation and psychotherapy (66–68) can be combined to form synergistic interventions. Vestibular rehabilitation enhances compensatory capacity through balance training, while psychotherapy aids patients in coping with anxiety. However, both should not be performed during acute episodes to ensure therapeutic efficacy and safety. Although existing approaches cannot reverse established hearing loss, early intervention can significantly improve patients’ quality of life and maintain auditory function.

### 5.2 Management strategies for VM

The concept and diagnostic criteria for VM were established relatively late, with limited high-quality clinical studies currently available. Its triggering factors resemble those of other migraine types. At this stage, treatment for VM primarily prioritizes lifestyle adjustments to avoid triggers, escalating to pharmacological interventions when symptom improvement is insufficient. The principles of preventive therapy for VM align with those for migraine, commonly using calcium channel blockers (flunarizine), tricyclic antidepressants (amitriptyline, nortriptyline), *β*-blockers (propranolol, metoprolol), and antiepileptic drugs (valproate, topiramate) (69–71). Notably, the novel calcitonin gene-related peptide (CGRP) receptor antagonist rimegepant has demonstrated unique advantages in recent clinical trials, showing efficacy not only in alleviating acute-phase headache symptoms (with a 35% higher 2-h pain freedom rate versus placebo) but also achieving a 62% improvement rate for accompanying vertigo symptoms (72). During acute VM attacks, vestibular suppressants such as promethazine and dimenhydrinate are recommended to alleviate vertigo and vomiting symptoms. However, Randomized Controlled Trials (RCTs) on VM treatment indicate that flunarizine is superior to propranolol in preventing headaches and vestibular symptoms, with RCTs confirming its greater efficacy compared to betahistine or vestibular rehabilitation in reducing vertigo frequency and severity (71). Moreover, triptans show significant potential for application during acute-phase management (73, 74).

## 6 Discussion and conclusions

This review provides a comparative analysis of MD and VM across multiple dimensions, including pathogenesis, clinical manifestations, and diagnostic criteria. Although the two conditions exhibit significant symptomatic overlap—such as episodic vertigo, tinnitus, and aural fullness—leading to challenges in clinical differentiation, they are fundamentally distinct disease entities. MD is pathologically characterized by endolymphatic hydrops (EH), associated with disruptions in ionic homeostasis, immune-mediated inflammation, and specific genetic factors (e.g., EGFLAM). Its hearing loss is typically progressive, fluctuating, and irreversible. In contrast, VM originates from central nervous system hyperexcitability, involving ion channel defects, cortical spreading depression, and the release of neuropeptides such as CGRP. VM-related vertigo is often provoked by changes in head position, and any accompanying hearing loss is usually mild and reversible. Migraine features—including photophobia, phonophobia, and/or headache—are central to its presentation. At the molecular level, MD demonstrates a unique innate immune activation profile (e.g., a “monocyte-driven cluster” identified via single-cell studies), with potential biomarkers including CHMP1A and MMP9. VM, on the other hand, shares a type 1 innate immune polarization response with migraine, with CGRP being a key biomarker. Diagnostically, MD relies on the correlation between auditory and vestibular symptoms and evidence of EH, whereas VM diagnosis is based on the association between vestibular symptoms and migraine features. Treatment strategies also diverge: MD management follows a stepped-care approach (e.g., diuretics, intratympanic injections) aimed at controlling vertigo and preserving hearing, while VM treatment aligns with migraine prophylaxis strategies, emphasizing preventive medications such as flunarizine. In summary, current clinical, imaging, and molecular evidence supports the notion that MD and VM are independent disorders. The diagnostic challenge primarily stems from their symptomatic similarities and the fact that vertigo itself can trigger migraine attacks—often leading to reliance on clinicians’ subjective experience and perspective for differentiation. Therefore, future research should focus on: (1) utilizing single-cell transcriptomics and genetically engineered animal models to further elucidate disease mechanisms; (2) establishing imaging-based biomarkers via high-resolution inner ear MRI; (3) validating candidate serum biomarkers using standardized proteomic platforms; and (4) integrating clinical, imaging, and molecular markers through machine learning to improve diagnostic accuracy and enable personalized treatment strategies.
